# Atypical case of anterograde amnesia after cerebral infarction and anti-NMDA encephalitis post Covid 19 infection: A complex clinical case

**DOI:** 10.1192/j.eurpsy.2024.1075

**Published:** 2024-08-27

**Authors:** M. Gebele

**Affiliations:** Subacute psychiatry unit, Hospital Gintermuiza, Jelgava, Latvia

## Abstract

**Introduction:**

Only a few cases of primary anterograde amnesia with confabulation after severe complex brain damage have been described in the literature.

**Objectives:**

To describe a case of anterograde amnesia with confabulation in a patient with severe and extensive brain damage.

**Methods:**

case report

**Results:**

Case presentation: A 48-year-old male patient with a medical history of diabetes mellitus type II, hypertension, presented to a psychiatric clinic for the first time. He was admitted to the hospital due to the manifestation of disruptive aggressive behaviour, aimless wandering, and excessive, impulsive expenditure of financial resources. At the time of hospitalization and during the hospital stay, the patient exhibited a state of elevated mood and anterograde amnesia compounded by the presence of prominent confabulation, easily irritable mood with a tendency to conflict. No physical limitations.

Background: The patient is an active long-distance driver for 15 years. A year before hospitalization in psychiatric clinic, he was travelling to Moscow, he had episode of headache and unconsciousness after which hospitalization. Diagnosed with multiple infarcts of embolic origin in the right frontal lobes, both cortical and subcortical, on the right side at the level of the uncus, in the medial anterior parts of the right occipital lobe, on the left side in the insula and at the level of the capsula externa, in the anterior basal part of the left temporal lobe. After hemodynamic stabilization, he was repatriated to Latvia. Stationary positive SARS-CoV-2 PCR, O2 support therapy required.

The patient develops auditory and visual hallucinations, which do not correct on antipsychotic therapy. Lumbar puncture was performed, which showed positive anti-NMDA antibodies, magnetic resonance - autoimmune limbic encephalitis with damage to the gyrus cinguli of the insula cortex of both temporal lobes and the right subfrontal part with spread throughout the right temporal lobe, bilaterally in the mediobasal structures of the temporal lobes and the right thalamus with progressive changes. The patient receives immunomodulatory therapy, plasma exchange and immune globulin. Hallucinations decrease on the background of therapy. At discharge - moderate ataxia in the legs, disorientation in time, severe short-term memory disorders.

The patient in his mind lives like nothing has happened and the same life continues.

**Image:**

**Figure:**
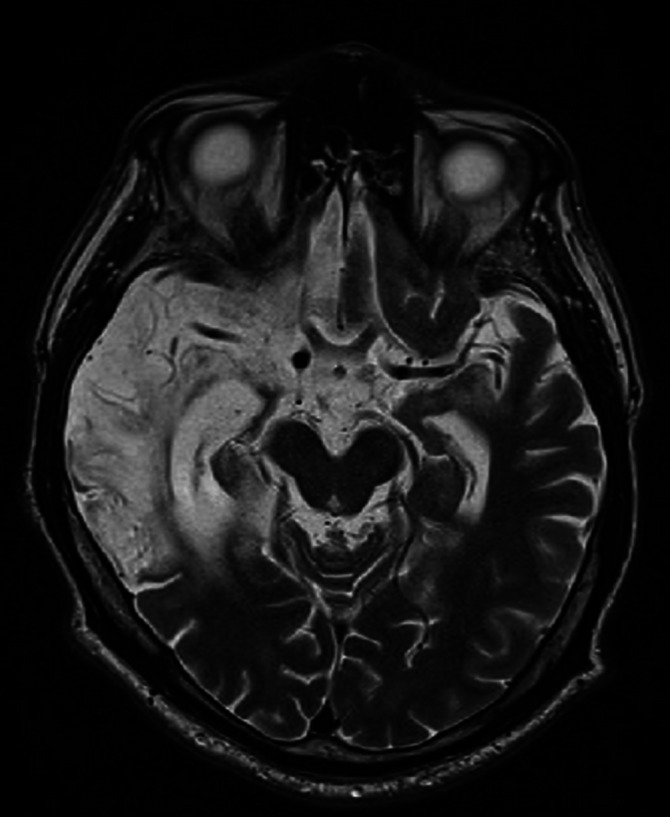

**Image 2:**

**Figure:**
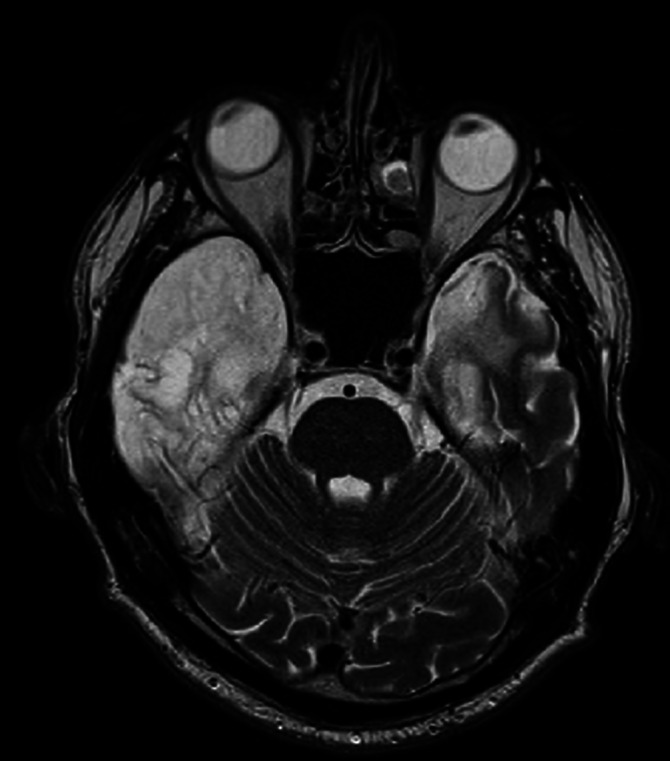

**Conclusions:**

The long-term prognosis for the patient remains uncertain, given the multifaceted nature of the condition and the extent of brain damage. Continuous monitoring, rehabilitation, and ongoing support will be essential to assess cognitive recovery and improve the patient’s quality of life.

**Disclosure of Interest:**

None Declared

